# Limb salvage using Viabahn stent grafts for femoral and popliteal artery disruptions following bilateral type III open femur fractures from crush injury

**DOI:** 10.1016/j.jvscit.2025.102024

**Published:** 2025-10-15

**Authors:** Autumn Bertch, Timothy Pansegrau

**Affiliations:** a University of North Dakota School of Medicine and Health Sciences, Grand Forks, ND; b Department of Cardiovascular Surgery, Sanford Medical Center, Bismarck, ND

**Keywords:** Endovascular repair, Femoral artery injury, Limb salvage, Popliteal artery disruption, Vascular trauma, Viabahn stent graft

## Abstract

This is a case of successful limb salvage using Viabahn stent grafts in a 47-year-old male with bilateral type III open femur fractures and traumatic disruption of the superficial femoral and popliteal arteries following a crush injury. Both femoral arteries were treated with endovascular repair using a total of four Viabahn endoprostheses (two per side), which restored lower extremity perfusion and avoided the need for open bypass. Open bypass was not feasible due to extensive venous trauma rendering the veins unsuitable for conduit harvest; Viabahn stents provided the only viable option for limb salvage. The patient recovered distal motor and sensory function with sustained graft patency at follow-up. This case highlights the potential role of Viabahn stent grafts as a minimally invasive alternative to autologous vein grafts for vascular trauma repair in complex, high-acuity settings.

The GORE Viabahn endoprosthesis with heparin bioactive surface is Food and Drug Administration-approved in the United States for improving blood flow in patients with symptomatic peripheral artery disease, including lesions in the iliac arteries, de novo and restenotic lesions of the superficial femoral artery (SFA), and stenosis or thrombotic occlusion at the venous anastomosis of synthetic arteriovenous access grafts.^1^ Although these indications are well-established, there is emerging interest in its application in vascular trauma. In one case, Kawatani et al described the use of endovascular Viabahn stent grafting for repair of a traumatic brachial artery injury after a crush injury.^2^ However, there are limited reports of cases describing the use of Viabahn stent grafts for repair of traumatic disruptions of the SFA and popliteal artery. This case contributes to the expanding body of literature by detailing the successful use of multiple Viabahn endoprostheses in the management of traumatic disruption of the bilateral SFAs and the left popliteal artery, secondary to bilateral femur fractures sustained in a crush injury. Written informed consent was obtained from the patient for publication of this case report and any accompanying images.

## Case report

**Fig 1:**
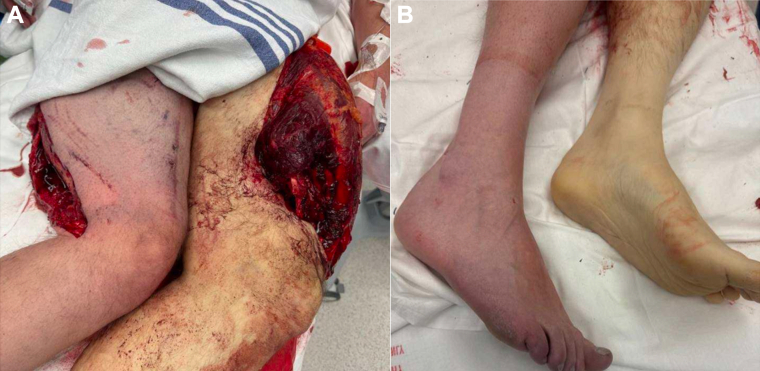
**(A)** Initial presentation demonstrating extensive bilateral lower extremity trauma with exposed femoral fractures and soft tissue disruption following crush injury. **(B)** Preintervention clinical image demonstrating differential perfusion between the bilateral lower extremities. The left foot appears well-perfused, whereas the right foot shows pallor and signs of acute limb ischemia prior to revascularization.

A 47-year-old male with no known past medical history presented to the Emergency Department after being crushed between a vehicle and a building. He sustained extensive bilateral lower extremity trauma, including bilateral type III open, displaced, comminuted femur fractures, a transected left SFA and popliteal vein, and an interval tear/dissection of the right SFA. Bilateral tourniquets were applied in the field due to uncontrolled arterial hemorrhage. Upon arrival to the Emergency Department, the patient was profoundly hypotensive from blood loss and received 11 units of packed red blood cells, four units of fresh frozen plasma, and one unit of platelets. On examination, the left lower extremity was pale, pulseless, monoplegic, and exhibited complete sensory loss, with no evidence of distal perfusion. The right lower extremity was dusky but demonstrated preserved strength, sensation, and faint distal pulses (Fig 1, *A* and *B*).

**Fig 2:**
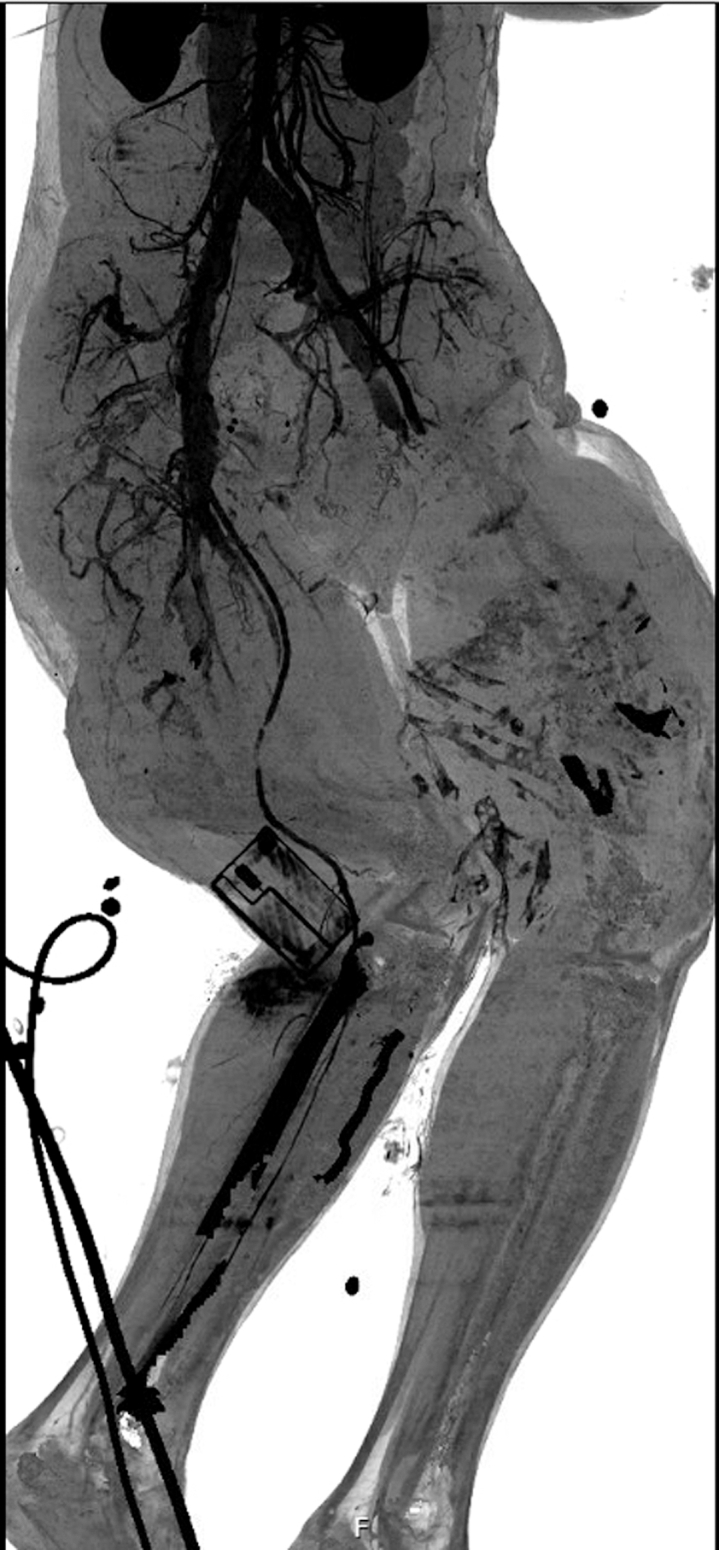
Initial computed tomography angiogram demonstrating abrupt loss of contrast opacification in the left superficial femoral (SFA) and popliteal arteries, consistent with complete vascular transection. This image confirms that the injury extended beyond the SFA into the popliteal segment, supporting the need for long-segment endovascular repair.

Computed tomography imaging of the head, cervical spine, chest, abdomen, pelvis, and bilateral lower extremity runoff was obtained (Fig 2). There were no acute findings in the head, cervical spine, or chest. However, computed tomograhy angiography of the abdomen and pelvis demonstrated left inferior and superior pubic rami fractures, bilateral open femur fractures, and a right popliteal artery injury. Due to continued hemorrhage when the left tourniquet was removed, it was reapplied prior to imaging, which limited visualization of the left SFA and distal vasculature. Orthopedic and vascular surgery were emergently consulted, and the patient was taken to the operating room for vascular repair and fracture stabilization.

**Fig 3:**
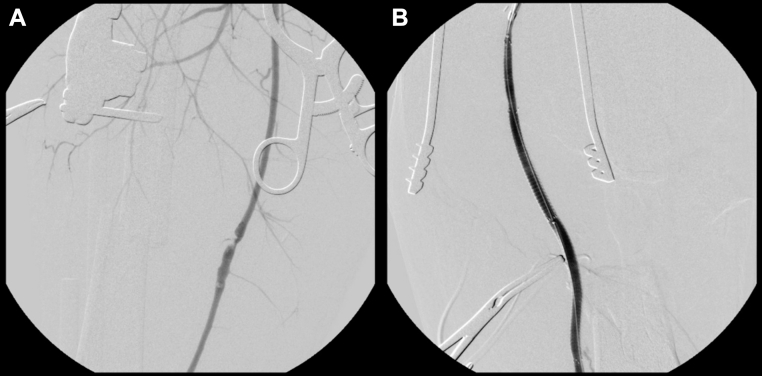
**(A)** Digital subtraction angiography of the left thigh demonstrating abrupt tapering and loss of contrast opacification in the superficial femoral artery (SFA), consistent with complete arterial transection. **(B)** Postintervention angiography showing restored inline flow through the Viabahn stent graft without evidence of contrast extravasation.

**Fig 4:**
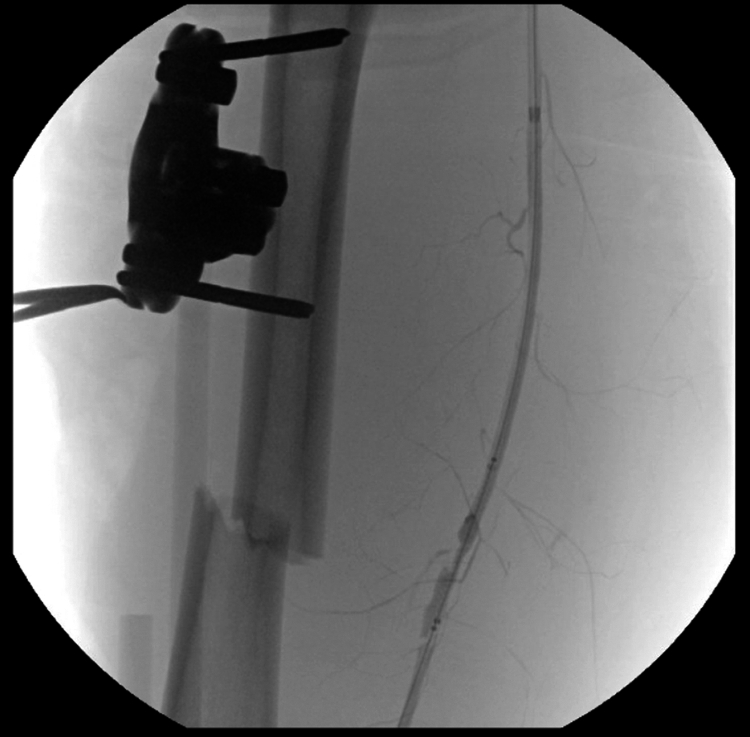
Fluoroscopic image demonstrating deployment of a Viabahn stent graft across the transected segment of the left superficial femoral artery (SFA). Radiopaque stent markers confirm accurate positioning.

Intraoperatively, the left thigh was explored through the open fracture site, revealing transection of the SFA and popliteal artery. The vessels showed poor backbleeding. A #4 Fogarty catheter was passed, yielding limited backbleeding but no clot retrieval. The popliteal artery was dissected out several centimeters distal to the site of the injury, revealing approximately 10 cm of arterial loss. Due to the complete transection, wire passage was achieved by directly puncturing the SFA proximal to the injury. A guidewire was carefully passed through the open arterial ends to the distal vessel under direct visualization. The stents were subsequently deployed over the guidewire to bridge the defect. A 7-French sheath and guidewires were introduced through the SFA and into the distal vessel, and an arteriogram confirmed distal flow. A 6 × 15 mm Viabahn graft was deployed but did not span the arterial defect adequately, so an additional 6 × 10 mm Viabahn graft was placed. Completion angiography showed adequate three-vessel runoff (Fig 3, *A* and *B* and Fig 4). The left popliteal vein was completely transected with an approximately 10-cm segmental loss, precluding the use of a vein graft for reconstruction. Orthopedic surgery performed irrigation and debridement and placed bilateral external fixators. Redundancy was left in the vascular grafts to accommodate limb length restoration.

On the right side, a femoral artery cutdown was performed. A guidewire was advanced into the distal SFA, and two 6 × 5 mm Viabahn grafts were placed to treat the dissection. Flow was restored, and a pursestring suture was used to secure the artery in the groin. The groin was then closed with multiple layers of Vicryl and Monocryl. The patient was transferred to the surgical intensive care unit intubated, on phenylephrine, and continued to receive fluid resuscitation.

On postoperative day 1, there was concern for evolving compartment syndrome, so the patient underwent an emergent medial and lateral longitudinal fasciotomy of the left lower extremity. Postoperatively, bilateral pedal pulses were present, and both legs were warm and well-perfused. By hospital day 2, the patient had regained distal motor and sensory function. Aspirin was initiated on hospital day 3. The patient was extubated on hospital day 4 but fell on day 5 due to alcohol withdrawal, reinjuring his left femur and requiring external fixator revision. He remained intubated postoperatively. Plastics was consulted on day 6 for complex soft tissue injuries. On day 8, he underwent fasciotomy closure, wound debridement, partial closure, and wound VAC placement. Dialysis began on day 11 for acute tubular necrosis with hepatorenal syndrome.

His course included transient hyperbilirubinemia (peak, 15 mg/dL) without biliary obstruction, resolving spontaneously. He remained in the intensive care unit on and off with vasopressors and dialysis until transfer to the floor on day 15. On day 19, he underwent open reduction and internal fixation and fixator removal of the left femur. Tube feeds were utilized until the final week of his admission due to aspiration risk. The patient completed his antibiotic course on hospital day 26 and was discharged to a long-term acute care facility on aspirin 81 mg and clopidogrel 75 mg daily. He was scheduled for outpatient follow-up with vascular surgery, plastic surgery, and orthopedics.

At 2-month follow-up in the vascular surgery clinic, the patient demonstrated warm, well-perfused feet with palpable distal pulses bilaterally. The only long-term sequela of his injury was persistent left-sided foot drop. He was advised to continue dual antiplatelet therapy, perform routine foot checks, and follow-up annually for vascular surveillance.

## Discussion

Severe lower extremity trauma commonly results in concomitant femoral and popliteal vascular injury, particularly in cases involving open femur fractures. Although overall incidence remains relatively low—approximately 1.6% in adult trauma patients and 0.6% in pediatric populations—rates are significantly higher in military or combat settings, ranging from 6% to 12%. Specifically, SFA injury associated with femur fractures occurs in approximately 1% to 2% of young, otherwise healthy trauma patients. Injuries to the popliteal artery, tibioperoneal trunk, or trifurcation vessels occur in 1.5% to 2.8% of all tibial fractures, increasing to nearly 10% in open fractures. Additionally, popliteal artery injuries requiring surgical repair are seen in up to 16% to 20% of knee dislocations.^3^

Current guidelines, such as the 2025 European Society for Vascular Surgery Clinical Practice Guidelines, support the use of short synthetic interposition grafts as a Class I recommendation for emergency vascular reconstruction.^4^ However, there are no formal guidelines regarding the use of longer endovascular stent grafts, such as the Viabahn, in traumatic vascular repair. Despite this, the Viabahn stent graft is increasingly being utilized in trauma scenarios due to several practical advantages over traditional transpositional vein grafts. Unlike open surgical grafts, the Viabahn is deployed endovascularly, allowing for quicker revascularization and less disruption to surrounding soft tissue—critical in polytrauma patients or in contaminated wounds. Its flexibility and ability to be placed with redundancy make it particularly well-suited for managing vascular injuries in conjunction with orthopedic manipulation or limb length restoration.

Additionally, the Viabahn eliminates the need for autologous vein harvest, preserving native veins for future use and avoiding complications related to additional surgical sites. Although the Viabahn stent graft is a known endovascular tool, its use in this case highlights a unique and innovative application in the setting of extensive bilateral lower extremity trauma. Open autologous vein bypass—typically the gold standard in young patients—was not feasible due to the severity of venous injury and the absence of viable conduit. The patient’s lower extremities were severely injured, and endovascular repair with Viabahn stents represented the only viable option for achieving timely revascularization and limb salvage. This case demonstrates the critical utility of endovascular grafting in high-acuity trauma when traditional techniques are not possible. Multiple Viabahn stents can also be overlapped to span long or complex injuries—something not easily achieved with standard vein grafts. Although long-term durability data in trauma settings remains limited, the immediate benefits of speed, accessibility, and adaptability make the Viabahn a valuable tool in the acute setting.

## Conclusions

This case illustrates the successful use of Viabahn stent grafts in the emergent management of complex bilateral lower extremity vascular injuries. The patient experienced restoration of perfusion and function, without early complications, following endovascular repair with multiple Viabahn endoprostheses.

Although current guidelines have yet to endorse the use of Viabahn stent grafts explicitly, this and similar cases support their role as a viable alternative to traditional vein grafts, especially in high-acuity scenarios where rapid intervention is essential. Compared with open transpositional grafting, Viabahn stents offer a minimally invasive, time-efficient solution with advantages in soft tissue preservation, procedural speed, and anatomical flexibility. As the body of evidence continues to grow, endovascular stent grafts like the Viabahn may become integral to vascular trauma protocols—particularly in settings where traditional grafting is not ideal. Further research and long-term outcomes will be crucial in guiding future practice and establishing formal clinical recommendations.

## Declaration of generative AI and AI-assisted technologies in the writing process

During the preparation of this work the authors used ChatGPT (OpenAI) to improve readability and language of the manuscript. After using this service, the authors reviewed and edited the content as needed and take full responsibility for the content of the publication.

## Funding

None.

## Disclosures

None.
